# Dysregulation of microRNA Expression and Function Contributes to the Etiology of Fetal Alcohol Spectrum Disorders

**DOI:** 10.35946/arcr.v35.1.03

**Published:** 2013

**Authors:** Sridevi Balaraman, Joseph D. Tingling, Pai-Chi Tsai, Rajesh C. Miranda

**Affiliations:** **Sridevi Balaraman, Ph.D.**, *is a postdoctoral research associate, in the Department of Neuroscience and Experimental Therapeutics, Texas A&M Health Science Center, Bryan, Texas.*; **Joseph D. Tingling**, *is a graduate research assistant, in the Department of Neuroscience and Experimental Therapeutics, Texas A&M Health Science Center, Bryan, Texas.*; **Pai-Chi Tsai**, *is a graduate research assistant, in the Department of Neuroscience and Experimental Therapeutics, Texas A&M Health Science Center, Bryan, Texas.*; **Rajesh C. Miranda, Ph.D.**, *is a professor in the Department of Neuroscience and Experimental Therapeutics, Texas A&M Health Science Center, Bryan, Texas.*

**Keywords:** fetal alcohol spectrum disorders, fetal development, microRNAs, cellular regulation, genetic factors, epigenetic factors, environmental factors, animal models, cell culture studies

## Abstract

MicroRNAs (miRNAs) are members of a large class of non–protein-coding RNA (ncRNA) molecules that represent a significant, but until recently unappreciated, layer of cellular regulation. Assessment of the generation and function of miRNAs suggests that these ncRNAs are vulnerable to interference from genetic, epigenetic, and environmental factors. A small but rapidly expanding body of studies using a variety of animal- and cell culture–based experimental models also has shown that miRNAs are important targets of alcohol during fetal development and that their dysregulation likely plays a significant role in the etiology of fetal alcohol spectrum disorders (FASD). Accordingly, an analysis of the regulation and function of these miRNAs may yield important clues to the management of FASD.

MicroRNAs (miRNAs) are members of a vast, evolutionarily ancient, but poorly understood class of regulatory RNA molecules, termed non–protein-coding RNAs (ncRNAs). This means that in contrast to RNA molecules generated during gene expression (i.e., messenger RNA [mRNA] molecules), they are not used as templates for the synthesis of proteins. ncRNAs are encoded within the genomes of both eukaryotic and prokaryotic organisms and represent a novel layer of cell regulation and function that rivals the diversity and function of protein-coding mRNAs (for review, see Mattick 2007).

In recent years, researchers have investigated whether, and how, miRNAs interact with beverage alcohol (i.e., ethanol) and/or mediate its effects. Initial studies (Sathyan et al. 2007) explored the ethanol–miRNA interactions in fetal neural stem cells. Since then, increasing evidence has indicated that miRNAs play a role in the etiology of alcoholism (Pietrzykowski et al. 2008) and potentially alcohol withdrawal (Guo et al. 2011), as well as in ethanol’s effects on brain development (Guo et al. 2011; Tal et al. 2012; Wang et al. 2009), brain damage associated with adult alcoholism (Lewohl et al. 2011), and liver damage (i.e., hepatotoxicity) (Dolganiuc et al. 2009; Tang et al. 2008). Other drugs of abuse such as nicotine are also known to influence miRNA expression (Huang 2009); furthermore, ethanol and nicotine collaborate to regulate the expression of miRNAs in neural tissues (Balaraman et al. 2012). These data collectively suggest that miRNAs are an important, but as yet poorly understood, component of alcoholism and ethanol-associated toxicology and damage to the developing fetus (i.e., teratology). This review specifically focuses on the association between miRNAs and the developmental effects of ethanol exposure, examining both the current data and future potential for research in this field of ncRNA biology to promote a coherent understanding of teratology associated with alcohol exposure.

## Fetal Alcohol Spectrum Disorders

Maternal alcohol consumption during pregnancy can lead to a constellation of brain, face, cardiovascular, and skeletal defects of varying severity that collectively have been termed fetal alcohol spectrum disorders (FASD). At the extreme end of the spectrum of severity is fetal alcohol syndrome (FAS) (Clarren 1986), which is characterized by craniofacial abnormalities (e.g., small openings of the eyes, thin upper lip, flattened area above the upper lip), motor dysfunction, impaired coordination of muscle movements (i.e., ataxia), behavioral disturbances, and cognitive deficits as well as growth retardation (Jones et al. 1973). According to the Centers for Disease Control and Prevention, the incidence of FAS is 1 to 3 per 1,000 live births, and these rates increase to 10 to 15 per 1,000 in at-risk groups, such as the foster care population (May and Gossage 2001). More recent estimates suggest that the prevalence of FASD in school-aged children in the United States is between 2 and 5 percent (May et al. 2009). FASD imposes significant socioeconomic costs on families and society. The lifetime cost of caring for a child with FASD was estimated at about $2 million, and the total annual cost of FASD in the United States was estimated at $4 billion in 2004 (Lupton et al. 2004); these costs may be significantly higher today.

Although the facial characteristics seen in patients with FASD are the most obvious signs of fetal alcohol exposure, the most devastating consequences of prenatal alcohol exposure are brain defects that result in cognitive, affective, and motor deficits (Sampson et al. 2000). Therefore, understanding the diverse effects of alcohol on the developing brain during pregnancy may provide researchers with the key to developing therapies for managing both fetal and adult effects of alcohol exposure during pregnancy. This review focuses on an emerging body of data from animal and cell-culture studies that implicates miRNA dysregulation in the etiology of FASD.

## Focus on miRNAs

miRNAs are a class of ncRNAs that posttranscriptionally regulate the expression of protein-coding genes. When protein-coding genes are expressed (i.e., the encoded protein is produced), first an mRNA copy of the corresponding DNA sequence is generated in a process called transcription. This mRNA molecule consists of three parts: a noncoding start region (i.e., the 5′-end), the sequence actually containing the information for the encoded protein (i.e., the open reading frame), and a noncoding tail region (i.e., the 3′-end). miRNAs mainly act by binding to the 3′-untranslated region of their mRNA targets (Ambros et al. 2003; Bartel 2004; Ghildiyal and Zamore 2009), although that is not the only function attributable to these molecules. Many microRNAs are evolutionally conserved across species. They initially were discovered in the roundworm *Caenorhabditis elegans* (Lee et al. 1993), but since then they also have been found in plants, invertebrates, mammals, and humans (Bartel 2009). miRNAs play crucial roles in development, stem-cell self-renewal, programmed cell death (i.e., apoptosis), and cell-cycle regulation but also feature prominently in human disease, including cancers and neurodegenerative and metabolic diseases (Ambros 2004; Bartel 2004). miRNAs are abundant in the central nervous system (CNS) (Krichevsky et al. 2003; Vreugdenhil 2010), and brain miRNAs are crucial for regulating nerve cell generation (i.e., neurogenesis); neuronal degeneration; and maintaining normal neuronal functions associated with memory formation, neuronal differentiation, and synaptic plasticity (Li 2010; Schratt 2009; Smalheiser 2009).

### miRNA Biogenesis

**Figure 1:**
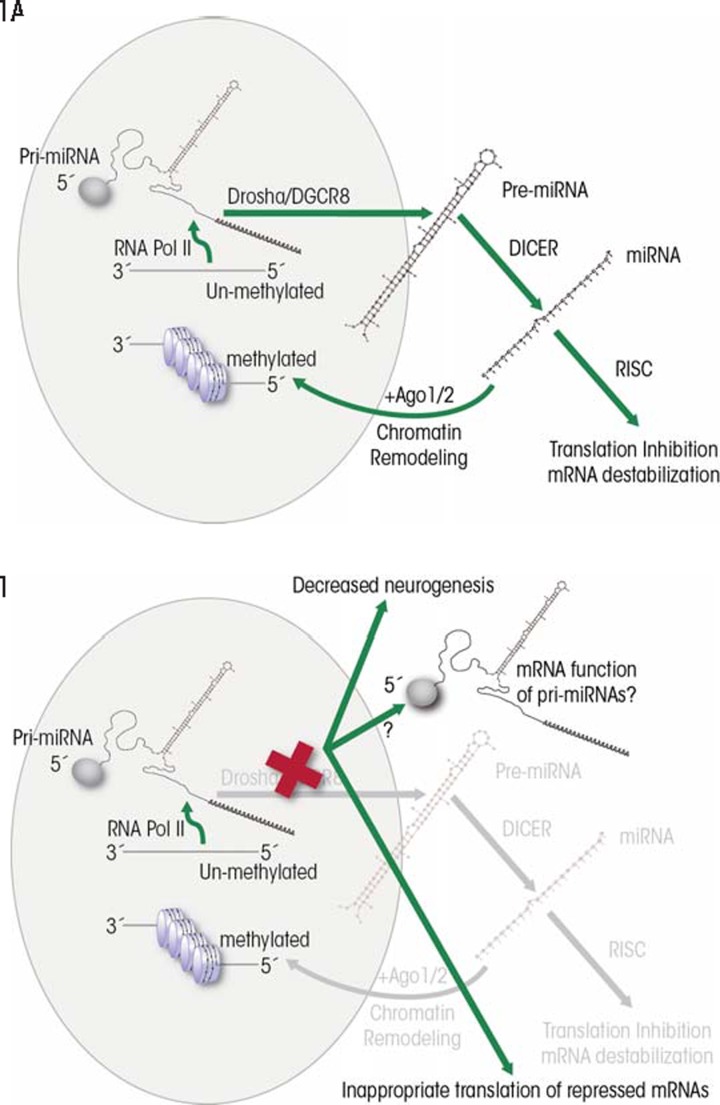
Models for standard (i.e., canonical) and disturbed (i.e., noncanonical) modes of miRNA biogenesis and function. **(A)** miRNAs often are generated (i.e., transcribed) from miRNA genes, as long mRNA-like transcripts, with a “cap” at the start (i.e., 5′-end) and several adenosine units at the end (i.e., 3′- polyA tail). The initial primary miRNA transcripts (pri-miRNAs) are processed to shorter, hairpin-shaped premature miRNAs (pre-miRNAs) by a protein complex called the DiGeorge syndrome critical region-8 (Drosha/DGCR8) complex. The pre-miRNAs then are transported to the cytoplasm for final processing into mature miRNAs by the Dicer complex. Mature miRNAs attain their function by being integrated into RNA-induced silencing complexes (RISC) that can degrade target mRNAs or silence translation. Processed miRNAs also can relocate to the nucleus to influence chromatin remodeling. **(B)** Disturbances in Drosha/DGCR8 processing (e.g., because of a mutation in the genes encoding these enzymes) may reveal alternate, mRNA-like functions of unprocessed pri-miRNAs and result in disrupted stem cell maturation.

**Figure 2:**
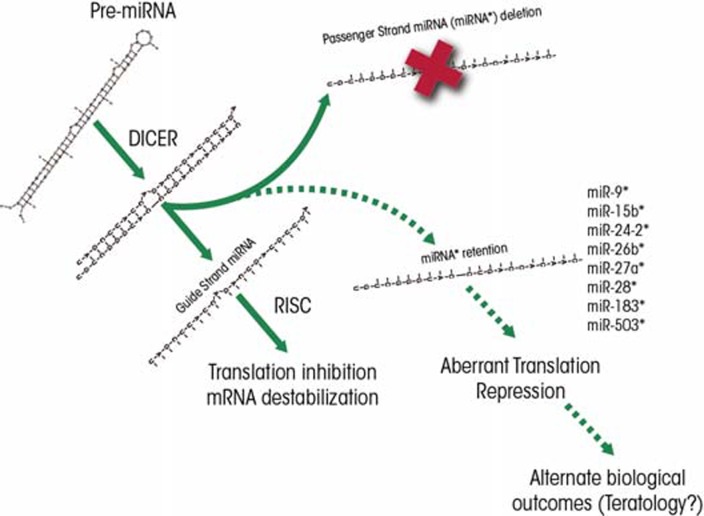
Model for the activity of the two strands of the processed pre-miRNA molecules (i.e., the guide strand [miRNA] and passenger strand [miRNA*]). Dicer processing of pre-miRNAs typically results in the formation of a guide stand miRNA that binds to the RNA-induced silencing complex (RISC). This guide strand can be derived from either the 3′- (termed −3p) or 5′- (termed −5p) end of the pre-miRNA. The complementary passenger strand typically is degraded. However, under various conditions, including ethanol exposure, miRNA* strands may be retained or otherwise differentially regulated, resulting the emergence of alternate biological end points. SOURCE: Guo et al. 2012, Acer 2012, Tal et al. 2012.

miRNAs are encoded within the genome either as independent genes or in gene clusters; however, they also can be encoded within introns^1^ of protein-coding genes, or even within introns and exons of another type of ncRNA called long intergenic non-(protein)-coding RNAs (lincRNAs). The generation of mature miRNAs from these coding sequences is a multistep process, as follows (see figure 1A):

- A normal transcription process, which is mediated by an enzyme called RNA-polymerase II, generates a longer primary transcript termed pri-miRNA. Like mRNA, the pri-miRNA transcripts can have certain modifications at their ends (i.e., a “cap” at the 5′-end and multiple adenosine units [i.e., a poly-A tail] at the 3′-end) and can be spliced (Cai et al. 2004). Further -more, the pri-miRNAs typically are folded into a double-stranded, hairpin–loop structure several hundred base pairs in length.
- Most pri-miRNA transcripts are processed within the nucleus by a protein complex called the DiGeorge syndrome critical region-8 (Drosha/DGCR8) “microprocessor” complex to generate stem-loop structures termed pre-miRNAs that are approximately 70 nucleotides in length (Han et al. 2006; Lee et al. 2003).
- The pre-miRNAs are moved from the nucleus to the cytoplasm by a chaperone protein called exportin-5 (Bohnsack et al. 2004).
- Within the cytoplasm, a protein complex known as Dicer enzyme further processes pre-miRNAs into mature double-stranded miRNA molecules (Hutvagner et al. 2001; Zhang et al. 2002). This process, and thus miRNA formation in general, is crucial for embryonic development because mutations in the Dicer proteins, which are exclusively part of the miRNA processing machinery, cause death of the embryo (Bernstein et al. 2003).
- Once the Dicer complex is cut off to release the mature miRNA, one strand of the double-stranded molecule, termed the guide strand miRNA, preferentially attaches to another protein complex called RNA-induced silencing complex (RISC). This results in a microribonucleoprotein (miRNP) complex that can either destabilize mRNA transcripts or repress the next step of gene expression in protein-coding genes (i.e., translation) (Mourelatos et al. 2002; Williams 2008). The second, complementary strand, known as passenger strand or miRNA* (see figure 2) has been thought to be quickly degraded (Matranga et al. 2005). However, as discussed later in this article, recent studies indicate that passenger-strand miRNAs can be retained by cells and exhibit independent biological functions.
- Finally, the mature miRNA can be degraded by an enzyme called 5′-3′ exoribonuclease (XRN2) (Bail et al. 2010).

### Role of miRNAs in Ethanol’s Teratologic Effects

In 2007, Sathyan and colleagues (2007) showed for the first time that miRNAs could mediate the effects of ethanol or indeed other teratogens. Using isolated tissue from the nervous system (i.e., neuroepithelium) of second-trimester fetuses, the investigators demonstrated that ethanol suppressed the expression of four miRNAs—miR-9, miR-21, miR-153, and miR-335—in fetal neural stem cells (NSCs) and neural progenitor cells (NPCs). The simultaneous suppression of miR-21 and miR-335 accounted for earlier observations (Prock and Miranda 2007; Santillano et al. 2005) that ethanol-exposed NSCs/NPCs are resistant to apoptosis, whereas the suppression of miR-335 explained the increase in NSC/NPC proliferation. Three of the four suppressed miRNAs target the mRNAs for two proteins called Jagged-1 and ELAVL2/HuB;^2^ accordingly, by suppressing the miRNAs, ethanol induced the expression of both target mRNAs. ELAVL2/HuB overexpression promotes neuronal differentiation (Akamatsu et al. 1999), and Jagged-1– induced proliferation establishes neuronal identity (Salero and Hatten 2007). These data collectively suggest that by interfering with miRNA function, ethanol may deplete the fetal pool of NSCs/NPCs and promote premature neuronal differentiation. More recently, Tal and colleagues (2012), using a zebrafish model, also showed that ethanol exposure during embryonic development suppressed the expression of miR-9 and miR-153. Importantly, these investigators demonstrated both behavioral and anatomical consequences of miRNA depletion. In particular, miR-153 depletion resulted in significantly increased locomotor activity in juvenile zebrafish, reminiscent of increased hyperactivity observed in children with FASD.

Other developmental ethanol exposure models also have indicated that ethanol alters the expression of several miRNAs. For example, Wang and colleagues (2009) showed that ethanol exposure during a period bracketing the end of the first trimester to the middle of the second trimester resulted in altered miRNA expression in brain tissue sampled near the end of the second trimester. In that study, ethanol induced a significant increase in the expression of two miRNAs (i.e., miR-10a and miR-10b), resulting in down-regulated expression of a protein called Hoxa1 in fetal brains. Other analyses had indicated that loss of Hoxa1 function (e.g., from familial Hoxa1 mutations) is associated with a variety of cranial defects and mental retardation (Bosley et al. 2007). This suggests that by suppressing translation of Hoxa1 and related genes, ethanol-mediated induction of miR10a/b may lead to similar defects.

Although the miRNAs identified by Wang and colleagues (2009) do not overlap with those identified by Sathyan and colleagues (2007) in NSCs/NPCs, miR-10a/b upregulation may have similar consequences for premature NSC differentiation. For example, miR-10a/b promotes the differentiation of cells from a type of nerve cell tumor (i.e., neuroblastoma cells) by suppressing translation of a protein called nuclear receptor corepressor-2 (NCOR2) (Foley et al. 2011). This effect is similar to the induction of Elavl2/HuB and Jagged-1.

Finally, Guo and colleagues (2011) assessed the effects of chronic intermittent ethanol exposure on cultured neuronal cells obtained from mouse cerebral cortex at gestational day 15, which is equivalent to the middle of the second trimester. The investigators found that ethanol induced several miRNAs in these cells. Interestingly, a prolonged period of withdrawal following the ethanol exposure resulted in a more than fourfold increase in the number of significantly regulated miRNAs, suggesting that withdrawal itself also may have a significant damaging effect on neuronal maturation in the developing fetal brain. Although these data were obtained from a cell-culture model, the implications of maternal binge drinking–withdrawal cycles on fetal miRNAs and their control over neural differentiation need further investigation.

### Effects of Coexposure to Ethanol and Other Drugs on miRNA Levels

Pregnant women who abuse ethanol also are likely to coabuse other drugs, such as nicotine (Substance Abuse and Mental Health Administration 2009). These other drugs also can affect miRNA levels. For example, Huang and colleagues (2009) demonstrated that nicotine induced expression of miR140* in a developmental model using the rat PC12 cell line. These effects may enhance or oppose those of ethanol. Thus, a recent study showed that ethanol and nicotine behaved as functional antagonists—that is, miRNAs that were suppressed by ethanol in fetal NSCs/NPCs were induced by nicotine exposure (Balaraman et al. 2012). Moreover, nicotine prevented the ethanol-mediated decrease in these miRNAs; this effect was pharmacologically mediated by a certain type of nicotine receptor (i.e., the nicotinic acetylcholine receptor). There is little generalized evidence as yet that drugs of abuse interact at the level of miRNAs to regulate cell function. Nevertheless these findings suggest that such an interaction is a real possibility, and the consequences for the teratologic effects of the drugs are likely to be significant.

## Teratogenic Implications of Altered miRNA Biogenesis, Cellular Localization, and Function

The data cited above show that ethanol alters the expression of several miRNAs at different developmental stages and that these alterations have consequences for fetal neural development and behavior. miRNA dysregulation is likely to influence teratogenesis by destabilizing the mRNAs of individual genes or gene networks. However, emerging evidence indicates that miRNA function also can be altered at several stages in the miRNA biogenesis pathway. Although to date such alterations are poorly understood, they may have important implications for teratology. The following represent four intriguing possibilities.

First, the presence of a 5′ cap and a 3′-polyA tail indicates that primary miRNA transcripts may have characteristics and function like regular mRNAs, and indeed evidence has been found for such a role (Cai et al. 2004). Although the conditions that permit the appearance of mRNA-like functionality are unclear, it is likely that interference with Dicer/DGCR8, which is essential for miRNA processing, can lead to the emergence of alternate functionality associated with pri-miRNA transcripts (see figure 1B). In this context, it is interesting to note that disruption of the DGCR8 locus is associated with mental retardation and that DGCR8 deletion interferes with the maturation of embryonic stem cells, causing them to aberrantly retain their ability to differentiate into different cell types (i.e., their pluripotency) while initiating differentiation (Wang et al. 2007). In this instance, the biology of stem cells seems to be intimately linked with the development of normal brain function.

Second, until recently, the complementary miRNA* strands (Hutvagner et al. 2001) were thought to be quickly degraded following Dicer cleavage of the double-stranded pre-miRNA molecule (Matranga et al. 2005). However, recent evidence (Ghildiyal et al. 2010; Okamura et al. 2009; Tyler et al. 2008) shows that these passenger strands also can be functional, acting on their own binding sites and regulating expression of their own sets of targets (figure 2). Thus, both strands of a premiRNA can be functional, each with a specific set of targets. The ratio of functional guide versus passenger strand miRNAs is regulated by an as-yet-unknown biology. Guo and colleagues (2011) identified several ethanol-sensitive miRNA* species that mainly were induced following ethanol exposure. Furthermore, other drugs of abuse, such as nicotine, also have been shown to induce the expression of a miRNA* (i.e., miR140*) (Huang and Li 2009). Alterations in the miRNA-to-miRNA*s ratio are likely to yield alternate biological outcomes that are particularly relevant to teratogenesis, as has been demonstrated in an analysis of the established ethanol-sensitive miRNA, miR-9. Tal and colleagues (2012) showed in their developmental zebrafish model that in addition to decreasing miR-9 (which now is also called miR-9-5p [www.mirbase.org, miRBAse Release 19]), ethanol produced a more modest decrease in the expression of miR-9* (now called miR-9-3p). The ratio of miR-9 to miR-9* is important for development and teratogenesis because these two miRNAs work together to regulate two molecules controlling neuronal differentiation. Thus, miR-9 levels influence the levels of a neuronal differentiation inhibitor called RE1 silencing transcription factor/neuron-restrictive silencer factor (REST), whereas miR-9* regulates its cofactor, coREST (Packer et al. 2008). Therefore, the simultaneous suppression of miR-9 and miR-9* may be expected to result in derepression of the REST/coREST complex and, consequently, inhibition of neuronal differentiation. On the other hand, preferential suppression of either miR-9 or miR-9* would be predicted to alter the ratio of REST to coREST, which has important and complex consequences for neural stem-cell renewal and altered lineage specification (Abrajano et al. 2009, 2010). Clearly, the involvement of passenger-strand miRNA biology in teratogenesis needs further investigation.

Third, in humans, about 6 percent of mature miRNAs undergo editing by the enzyme adenosine deaminase (Blow et al. 2006), resulting in alterations in either miRNA processing or miRNA efficiency (Kawahara et al. 2007; Yang et al. 2005). Furthermore, evidence suggests that edited miRNAs may exhibit different target specificity compared with their nonedited counterparts (Ekdahl et al. 2012). miRNA editing increases during brain development and may permit the emergence of new biological functions (e.g., a novel translational control of the development of nerve cell extensions [i.e., dendritogenesis]) (Ekdahl et al. 2012). These data collectively suggest that the role of miRNA editing in ethanol teratology warrants further exploration.

Finally, emerging evidence indicates that some mature miRNAs are transported back to the nucleus, where they mediate the formation of heterochromatin^3^ (Gonzalez et al. 2008). This observation suggests that miRNAs can directly influence the epigenetic landscape (figure 1A). Ethanol also alters the epigenetic landscape in differentiating fetal NSCs (Zhou et al. 2011), and the contributory role of nuclear miRNAs to this process is unknown. All of these modifications to miRNA biology represent novel and uninvestigated layers of regulatory processes that may have important consequences for cell and tissue differentiation and, consequently, teratogenesis.

## Implications for the Management of FASD

Despite strong evidence that maternal alcohol consumption during pregnancy leads to harmful effects on the fetus, a significant number of women continue to report drinking even into the third trimester of pregnancy. Therefore, early detection and management of fetal alcohol exposure remains an urgent public health concern, as does the development of approaches to ameliorate or prevent ethanol’s detrimental effects. The identification of miRNAs as ethanol targets presents one hope for the development of novel therapeutic programs. miRNAs have coevolved with their mRNA targets to orchestrate development. It is possible that miRNA-like drugs may be used to mitigate the effects of fetal ethanol exposure on the development of specific organs. The challenge will be to identify tissue-specific miRNAs that can be used to reprogram development. In this context, miRNAs such as miR-9 make intriguing therapeutic targets because they are fairly specific to neuronal cells (Leucht et al. 2008; Shibata et al. 2011; Smirnova et al. 2005). Evidence that ethanol-sensitive miRNAs also are sensitive to nicotine (Balaraman et al. 2012) suggests a promising and alternative, pharmacological approach to reprogramming fetal development following maternal ethanol exposure. Recent evidence suggests that pharmacologic approaches can indeed be used successfully in human populations, for example, to normalize cellular miRNA levels in neurological diseases such as multiple sclerosis (Waschbisch et al. 2011). Such an approach therefore may be similarly efficacious with FASD. Finally, Guo and colleagues (2011) have implicated DNA methylation as a mechanism for miRNA regulation, and Wang and colleagues (2009) demonstrated that folic acid administration could reverse ethanol’s effects on miRNAs. These data suggest that nutritional supplementation programs also may be an effective means towards ameliorating the effects of miRNA dysregulation. Research into miRNA involvement in fetal alcohol teratology is in its infancy. However, this research has significant potential for both uncovering principles underlying alcohol’s detrimental consequences and for developing novel strategies for the management of fetal alcohol effects.

## Footnotes

1 Genes encoding specific proteins typically comprise not only the DNA sequences that contain the building instructions for the resulting protein, but also noncoding sequences (i.e., introns). These introns are interspersed with the coding sequences (i.e., exons). During transcription, first an RNA copy of the entire gene, including introns and exons, is generated. This transcript is then processed by cutting out the intron sequences, to generate the final mRNA molecule. This process is known as splicing.

2 Jagged-1 binds to (i.e., is a ligand of) Notch receptor and ELAVL2/HuB is a neuron-specific RNA-binding protein.

3 Heterochromatin is a tightly packed complex of DNA and proteins in the cell nucleus in which the DNA is not easily accessible to other proteins and therefore generally is not expressed. In contrast, a more lightly packed DNA–protein complex (i.e., euchromatin) is more easily accessible for transcription enzymes.
